# Imbalanced cortisol concentrations in glycogen storage disease type I: evidence for a possible link between endocrine regulation and metabolic derangement

**DOI:** 10.1186/s13023-020-01377-w

**Published:** 2020-04-19

**Authors:** Alessandro Rossi, Chiara Simeoli, Mariacarolina Salerno, Rosario Ferrigno, Roberto Della Casa, Annamaria Colao, Pietro Strisciuglio, Giancarlo Parenti, Rosario Pivonello, Daniela Melis

**Affiliations:** 1 Department of Translational Medicine, Section of Pediatrics, University of Naples “Federico II”, Naples, Italy; 2 Dipartmento di Medicina Clinica e Chirurgia, Sezione di Endocrinologia, University of Naples “Federico II”, Naples, Italy; 3 Maternal-Infant Department, Pediatrics Unit, “San Pio” Hospital, Benevento, Italy; 4 Telethon Institute of Genetics and Medicine, Pozzuoli, Italy; 5 Department of Medicine, Surgery and Dentistry “Scuola Medica Salernitana”, Section of Pediatrics, University of Salerno, Via Salvador Allende, 43 84081 Baronissi (Salerno), Italy

**Keywords:** Cortisol, 11βHSD1, Cholesterol, Insulin-resistance, Autoimmune

## Abstract

**Background:**

Glycogen storage disease type I (GSDI) is an inborn error of carbohydrate metabolism caused by mutations of either the G6PC gene (GSDIa) or the SLC37A4 gene (GSDIb). Glucose 6-phosphate (G6P) availability has been shown to modulate 11β-hydroxysteroid dehydrogenase type 1 (11βHSD1), an ER-bound enzyme catalyzing the local conversion of inactive cortisone into active cortisol. Adrenal cortex assessment has never been performed in GSDI. The aim of the current study was to evaluate the adrenal cortex hormones levels in GSDI patients.

**Methods:**

Seventeen GSDI (10 GSDIa and 7 GSDIb) patients and thirty-four age and sex-matched controls were enrolled. Baseline adrenal cortex hormones and biochemical markers of metabolic control serum levels were analyzed. Low dose ACTH stimulation test was also performed.

**Results:**

Baseline cortisol serum levels were higher in GSDIa patients (*p* = 0.042) and lower in GSDIb patients (*p* = 0.041) than controls. GSDIa patients also showed higher peak cortisol response (*p* = 0.000) and Cortisol AUC (*p* = 0.029). In GSDIa patients, serum cholesterol (*p* = 0.000), triglycerides (*p* = 0.000), lactate (*p* = 0.000) and uric acid (*p* = 0.008) levels were higher and bicarbonate (*p* = 0.000) levels were lower than controls. In GSDIb patients, serum cholesterol levels (*p* = 0.016) were lower and lactate (*p* = 0.000) and uric acid (*p* = 0.000) levels were higher than controls. Baseline cortisol serum levels directly correlated with cholesterol (ρ = 0.65, *p* = 0.005) and triglycerides (ρ = 0.60, *p* = 0.012) serum levels in GSDI patients.

**Conclusions:**

The present study showed impaired cortisol levels in GSDI patients, with opposite trend between GSDIa and GSDIb. The otherwise preserved adrenal cortex function suggests that this finding might be secondary to local deregulation rather than hypothalamo-pituitary-adrenal axis dysfunction in GSDI patients. We hypothesize that 11βHSD1 might represent the link between endocrine regulation and metabolic derangement in GSDI, constituting new potential therapeutic target in GSDI patients.

## Background

Glycogen storage disease type I (GSDI) is an inborn disorder of carbohydrate metabolism caused by the deficiency of microsomal glucose-6-phosphatase (G6Pase) system. It is characterized by accumulation of glycogen and fat in the liver and kidneys. Two major subtypes of GSDI have been identified: GSDIa, which is caused by mutations in the gene encoding the G6Pase alpha (G6Paseα), and GSDIb, caused by mutations in the gene encoding the glucose 6-phosphate (G6P) translocase (G6PT), which transports G6P from cytoplasm to microsomes. G6Paseα is expressed in the liver, kidney and intestine, whereas G6PT is ubiquitous. The clinical and biochemical phenotype of GSDI includes fasting hypoglycaemia, hepatomegaly, lactic acidosis, hypertriglyceridemia, hypercholesterolemia and hyperuricemia; GSDIb is also associated with neutropenia and neutrophil dysfunction, resulting in recurrent infections and predisposition to inflammatory bowel disease (IBD) [1].

G6P availability has been shown to modulate 11β-hydroxysteroid dehydrogenase type 1 (11βHSD1) activity. In GSDIa, the G6P excess in the endoplasmic reticulum (ER) (due to G6Paseα deficiency) has been associated to increased 11βHSD1 activity, while in GSDIb the lack of G6P in ER (due to G6PT deficiency) has been associated to decreased 11βHSD1 activity [2].

**Fig. 1:**
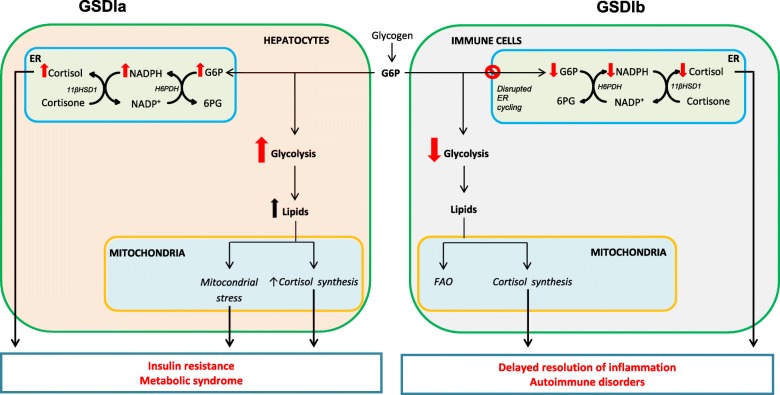
Proposed pathomechanism linking endocrine regulation and metabolic imbalance in GSDI. In GSDIa G6P accumulates in both cytosol and ER within the hepatocytes. Increased G6P availability in the ER upregulates 11βHSD1 activity resulting in increased cortisol regeneration. Increased G6P in the cytosol enhances glycolysis and lipid load to mitochondria resulting in mitochondrial stress and increased cortisol synthesis (secondary to increased substrate availability). Together, these secondary metabolic disturbances lead to increased risk of insulin-resistance and metabolic syndrome. In GSDIb G6PT defect results in disrupted ER cycling in immune cells (e.g. neutrophils, lymphocytes) and subsequently decreased cortisol regeneration with the ER and potentially reduced substrates to mitochondria for cortisol synthesis. Reduced cortisol availability might contribute to chronic inflammation and higher risk for autoimmune disorders. *G6P: glucose 6-phosphate, 6PG:6-phosphogluconactone, 11βHSD1:11β-hydroxysteroid dehydrogenase type 1, H6PDH: hexose-6-phosphate dehydrogenase, FAO: fatty acid oxidation*

11βHSD1 is an ER-bound enzyme catalyzing the conversion of inactive cortisone in active cortisol. It is typically expressed in glucocorticoid receptor-rich tissues, such as the liver, adipose tissue, lung and brain [3]. 11βHSD1 requires NADPH as a cofactor generated by the hexose-6-phosphate dehydrogenase (H6PDH)-mediated conversion of G6P to 6-phosphogluconactone (6PGL) [4]. The accumulation of G6P in ER fuels the G6PT-H6PDH-11βHSD1 system, leading to increased pre-receptorial activation of glucocorticoids [5]. Therefore, the G6PT-H6PDH-11βHSD1 system is crucial in the coupling between glucose metabolism and glucocorticoid response (see Fig. 1). Interestingly, in H6PDH knock-out mice a decreased negative feedback on the hypothalamo-pituitary-adrenal (HPA) axis has been observed [6].

Although an inverse correlation between serum cortisol concentrations and weight SDS has been demonstrated [7, 8], adrenal cortex assessment has never been performed in GSDI patients.

The aim of the current study was to evaluate adrenal cortex function in GSDI patients unveiling possible differences between GSDIa and GSDIb patients.

## Methods

### Subjects

The study protocol was in accordance with the Italian regulations on privacy protection and with the Helsinki Doctrine for Human Experimentation. All studies were performed after informed consent was obtained from adult subjects or the infants’ parents. Patients were recruited over a 12 months period. Seventeen GSDI patients (6 males and 11 females) were enrolled. Ten GSDIa patients (4 males and 6 females, mean age 12.11 ± 1.52, range 6–20 years) were compared to 20 age and sex matched controls. Seven GSDIb patients (2 males and 5 females, median age 14.90 ± 2.25, range 8–23 years) were compared to 14 age and sex matched controls. The diagnosis of GSDIa and GSDIb was based on mutation analysis of the G6PC and SLC37A4 gene, respectively. All patients were on dietary treatment. Each patient received uncooked cornstarch (UCCS), nocturnal gastric drip feeding (CNGF) or a combination of the two. Dietary regimens varied among different patients according to their families’ requests and attitudes.

Thirty-four subjects with normal random blood glucose and no history of hypoglycemia were included as healthy control participants. Each GSDIa or GSDIb patient was compared to two age and sex-matched controls.

### Clinical and biochemical parameters

The following clinical parameters were recorded: height, weight, body mass index (BMI), systolic and diastolic blood pressure (BP). Blood samples were obtained at 8 a.m. Fasting time ranged between 4 and 9 h. This was calculated according to patients’ usual fasting tolerance. 16/17 patients showed fasting tolerance between 4 and 6 h. One adult patient showed fasting tolerance of 9 h. To overcome the bias due to patients’ short fasting time the control subjects were asked to have blood and urine sampling after the same fasting time of his/her age and sex matched patient. Serum glucose, cholesterol, triglycerides (TG), lactate, uric acid and bicarbonate were assessed as markers of metabolic control. In order to control for possible interaction of cholesterol with triglycerides, Corrected Cholesterol (CChol) was also calculated as following: Cholesterol – (TG/5) [9].

### Hormonal studies

Fasting blood samples were obtained at 8 a.m. HPA axis function was assessed by evaluating adrenocorticotropic hormone (ACTH), cortisol, androstenedione, 17-hydroxyprogesterone (17OHP), dehydroepiandrosterone sulphate (DHEAS), renin, aldosterone serum levels as well as and 24-h Urinary Free Cortisol (UFC) levels using routine assays with commercially available kits. Cortisol, DEHAS, androstenedione, 17OHP were evaluated at baseline and after a low dose ACTH stimulation test using 1 μg Synacthen® (synthetic ACTH analogue). The timing of the ACTH stimulation test was arranged in order not to exceed patients fasting tolerance.

### Statistical analysis

“Peak cortisol” was defined as the maximum observed cortisol value measured following ACTH administration regardless of when it occurred. Area under the curve (AUC) was calculated by trapezoid formula. All data in the text or shown in the figures are expressed as mean ± SE. Statistical analysis was performed using Statistical Package for Social Science (SPSS 10 for Windows Update; SPSS Inc., Chicago, Illinois, USA). The comparisons between numerical variables were performed by Student’s t-test corrected for Fisher’s exact test. The normality of the distribution was checked by the Shapiro–Wilk test. One-way ANCOVA with Bonferroni-adjusted post hoc tests analysis was performed to control cortisol concentrations for covariates (cholesterol, triglycerides and CChol). Correlation study was performed by Spearman’s rank correlation. Cholesterol, TG and CChol were further assessed in multivariable linear regression analysis. The predictive capability of the multivariable regression model was checked by the F-test. Statistical significance was set at *p* < 0.05.

## Results

### Clinical and biochemical parameters (Table 1 and Additional file 1)

GSDIa patients showed increased cholesterol (*p* = 0.000), TG (*p* = 0.000), lactate (*p* = 0.000) and uric acid (*p* = 0.008) serum levels and reduced bicarbonate serum levels (*p* = 0.000) compared to controls. GSDIb patients showed reduced cholesterol (*p* = 0.016), CChol (*p* = 0.010) and bicarbonate (*p =* 0.002) serum levels and increased lactate (*p* = 0.000) and uric acid (*p* = 0.000) serum levels (*p* = 0.002) compared to controls. GSDIb patients showed lower height (*p* = 0.040) and height centile (*p* = 0.002) and weight centile (*p* = 0.030) than controls. Glucose concentrations ranged 4.4–7.8 mmol/L in GSDIa patients and 4.0–8.1 mmol/L in GSDIb patients (Additional file 1A). No significant difference in the remaining parameters was observed between GSDIa and GSDIb patients and controls.

**Table 1:** Clinical and biochemical markers of metabolic control in GSDI patients and control subjects

	GSDIa		Controls		GSDIb		Controls			
	Mean	SE	Mean	SE	Mean	SE	Mean	SE	Ia vs C	Ib vs C
**Age** (years)	12.10	1.52	11.90	1.00	14.90	2.25	15.18	1.59	0.909	0.922
**Fasting time** (hours)	5.20	0.46	5.65	0.27	5.29	0.20	5.79	0.24	0.418	0.200
**Height** (cm)	139.00	5.80	144.70	4.00	143.00	4.00	155.00	3.40	0.420	**0.040**
**Height** (centile)	20.10	9.00	40.80	4.90	20.70	7.50	56.80	4.40	0.080	**0.002**
**Weight** (Kg)	46.80	6.60	49.70	4.40	54.90	7.40	61.70	5.20	0.710	0.460
**Weight** (centile)	68.00	7.70	72.00	4.10	75.70	6.60	87.80	1.20	0.610	**0.030**
**BMI** (Kg/m2)	22.93	1.30	23.05	10.80	25.90	2.12	25.00	1.44	0.947	0.734
**BMI** (centile)	88.80	3.20	88.80	2.20	92.00	2.40	91.90	2.03	0.811	0.520
**Systolic BP** (mmHg)	104.50	3.11	98.00	2.25	103.30	3.14	112.50	3.66	0.104	0.121
**Diastolic BP** (mmHg)	69.00	1.94	65.00	1.80	64.71	1.78	66.79	1.45	0.132	0.400
**Glucose** (mmol/L)	5.14	0.32	4.76	0.07	5.91	0.56	5.09	0.14	0.113	0.080
**Cholesterol** (mmol/L)	4.95	0.29	3.86	0.13	2.70	0.15	0.22	8.62	**0.000**	**0.016**
**Triglycerides (TG)** (mmol/L)	4.28	0.63	1.00	0.09	1.31	0.32	1.22	0.12	**0.000**	0.757
**CChol** (mmol/L)	4.09	0.20	3.66	0.12	2.44	0.11	3.33	0.21	0.090	**0.010**
**Lactate** (mmol/L)	2.16	0.15	1.33	0.05	3.26	0.67	1.35	0.06	**0.000**	**0.000**
**Uric acid** (μmol/L)	303.37	17.62	227.23	16.64	367.11	33.54	225.19	14.00	**0.008**	**0.000**
**Bicarbonate** (mmol/L)	22.40	0.71	26.31	0.43	20.77	1.14	24.57	0.48	**0.000**	**0.002**

*BP* blood pressure, *CChol* corrected cholesterol

### Hormonal studies

**Fig. 2:**
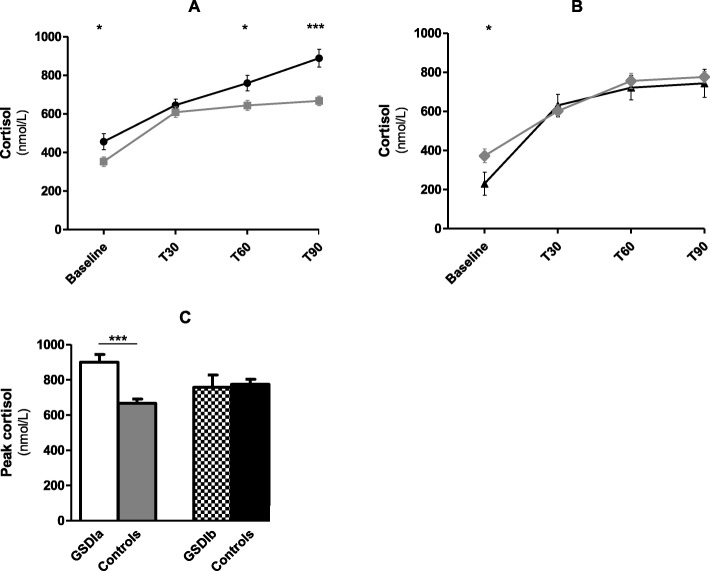
**a** Baseline and ACTH-stimulated cortisol levels in GSDIa patients (●) and controls (■). **b** Baseline and ACTH-stimulated cortisol levels in GSDIb patients (▲) and controls (◆). **c** Peak ACTH-stimulated cortisol levels in GSDIa and GSDIb patients and controls. * *p* < 0.05, *** *p* < 0.001. *T30: 30 min after ACTH analogue administration, T60: 60 min after ACTH analogue administration, T90: 90 min after ACTH analogue administration*

Baseline serum hormone levels and UFC are shown in Table 2 and Additional file 1. Serum cortisol levels were higher in GSDIa patients (*p* = 0.042, Fig. 2a) and lower in GSDIb patients (*p* = 0.041, Fig. 2b) than controls. GSDIa patients showed higher 60 min (*p* = 0.019, Fig. 2a) and 90 min (*p* = 0.000, Fig. 2a) cortisol levels after ACTH stimulation and higher peak cortisol response (p = 0.000, Fig. 2c) as well as cortisol area under the curve (AUC) (21,536 ± 884 vs 18,716 ± 764, *p* = 0.029) than controls. No significant difference in the remaining serum hormone levels, AUC and UFC were observed between GSDIa or GSDIb patients and controls. After controlling for covariates, no significant difference in 30 min and 60 min cortisol levels was observed between patients and controls (GSDIa: *p* = 0.645, GSDIb: *p* = 0.850); 90 min cortisol levels were significantly higher in GSDIa patients than controls (*p* = 0.007).

**Table 2:** Baseline hormone serum levels in GSDI patients and control subjects

	GSDIa		Controls		GSDIb		Controls		Significance (p)		Reference range
	Mean	SE	Mean	SE	Mean	SE	Mean	SE	Ia vs C	Ib vs C	
**ACTH** (pmol/L)	6.28	1.73	5.40	0.55	7.15	2.24	5.31	0.42	0.545	0.282	2.2–11.0
**Cortiso**l (nmol/L)	455.44	41.74	352.27	19.30	230.22	59.37	372.56	35.47	**0.042**	**0.041**	< 15 years: 83–580 > 15 years:220–525
**Androstenedione** (nmol/L)	1.16	0.33	1.44	0.23	2.24	0.28	2.28	0.35	0.493	0.944	Depending on Tanner stage
**17OHP** (nmol/L)	1.37	0.25	1.09	0.13	2.07	0.53	1.38	0.11	0.276	0.108	Depending on Tanner stage
**DHEAS** (nmol/L)	3392	1255	3496	272	4195	1755	3068	286	0.938	0.549	Depending on Tanner stage
**Renin**^**a**^ (pmol/L)	0.14	0.04	0.18	0.01	0.20	0.05	0.16	0.01	0.611	0.478	< 5 years: 0.07–0.21 > 5 years: 0.06–0.08
**Aldosterone**^**a**^ (pmol/L)	25.42	6.53	25.53	1.11	17.64	5.46	24.12	1.27	0.750	0.432	< 15 years: 1.80–28.80 > 15 years: 2.50–11.00
**UFC** (μg/24 h)^b^	55.83	8.02	65.30	5.72	81.29	47.94	62.67	10.26	0.360	0.610	1–10 years: 2–27 11–20 years:5–55 > 20 years: 20–90

^**a**^ 7 GSDIa and 6 GSDIb patients

^b^ 5 GSDIa and 3 GSDIb patients

*ACTH* adrenocorticotropic hormone, *17OHP*17-hydroxyprogesterone, *DHEAS* dehydroepiandrosterone sulphate, *UFC* 24-h urinary free cortisol

### Correlation study

**Fig. 3:**
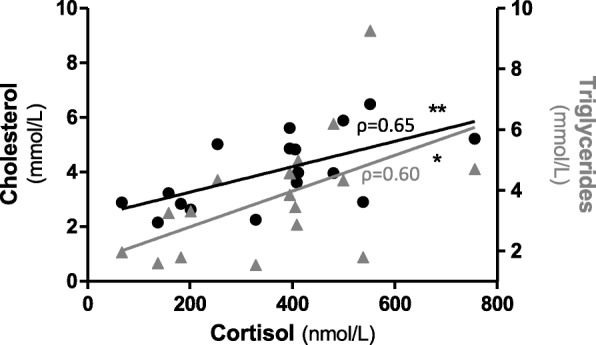
Correlation between baseline cortisol levels and cholesterol (●, ρ = 0.65, *p* < 0.01) and triglycerides (▲, ρ = 0.60, p < 0.05) levels in GSDI patients. * *p* < 0.05, **p < 0.01, *** *p* < 0.001

Baseline cortisol serum levels directly correlated with cholesterol (ρ = 0.65, *p* = 0.005) and TG (ρ = 0.60, *p* = 0.012) serum levels in GSDI patients (Fig. 3). A direct correlation between cholesterol and triglycerides was found (ρ = 0.77, *p* = 0.000). Multivariate analysis (F-test, *p* = 0.031) showed no significance for cholesterol (β = 0.50, *p* = 0.149), TG (β = 0.32, *p* = 0.640) and CChol (β = 0.39, *p* = 0.150).

## Discussion

An endocrine involvement has been extensively reported in GSDI [7, 8, 10–12]. Interestingly, most of the typical findings in GSDI (short stature, delayed puberty, hypothyroidsm, polycystic ovaries, osteoporosis) are similar to those of Cushing’s syndrome, suggesting a possible impairment in glucocorticoid metabolism in GSDI. To the best of our knowledge, systematic adrenal cortex assessment has never been performed in GSDI. In order to gather information on the function of the adrenal cortex, data concerning adrenal cortex hormones (both at baseline and after ACTH challenge) were collected in GSDIa and GSDIb patients. GSDIa patients showed higher baseline and ACTH-stimulated cortisol levels with GSDIb patients showing decreased baseline cortisol levels. The opposite cortisol profile between GSDIa and GSDIb points to a possible role of the metabolic defect *per se* in the endocrine imbalance. The results of the current study suggest that imbalanced cortisol levels in GSDI might be due to local deregulation rather than HPA axis activation. Cortisol role as a counter-regulatory hormone in glucose homeostasis should also be taken into account. No patient showed low blood glucose concentrations in the present study. Two GSDIb patients showed glucose concentration slightly above 4.0 mmol/L (Additional file 1A). Notably, GSDIb patients showed lower cortisol levels than controls in the present study. Glucose concentrations were not routinely measured at the end of the ACTH stimulation test based on the following considerations: 1) the timing of the ACTH stimulation test was arranged in order not to exceed patients fasting tolerance and 2) the administration of ACTH stimulates the release of cortisol from the adrenal cortex and no glucose lowering effect was expected. Indeed, data on glucose concentration at the end of the ACTH stimulation test available in four patients showed a relatively stable trend (Additional file 2). No correlation was found between glucose concentrations and cortisol levels at the end of the ACTH stimulation test in those patients (*p* = 0.800) suggesting that glucose concentration likely did not affect cortisol levels in the present study.

The regulation of adrenal cortex function is under control of HPA axis [13]. Nonetheless, 11βHSD1 has recently emerged as a local regulator mechanism [4]. An important biological function of liver 11βHSD1 (different from tissue-specific pre-receptoral metabolism) is a systemic shift of the cortisol:cortisone equilibrium towards active cortisol promoting the crucial metabolic and circulatory effects of cortisol [14]. Glucocorticoid excess is known to cause obesity and diabetes [15]. The considerable similarities between Cushing’s syndrome and metabolic syndrome (MS) have driven investigations on possible pathogenic role of glucocorticoids. Among all possible determinants (e.g. HPA axis, intracellular receptors density, prereceptorial metabolism), 11βHSD1 has emerged as the most plausible mechanism [16, 17]. The hepatic 11βHSD1 plays a key role in the development of MS [18, 19]. Conversely, 11βHSD1 knock-out mice are resistant to the development of MS [20, 21]. 11βHSD1 is nowadays a promising therapeutic target and a number of 11βHSD1 inhibitors are in development as potentially effective in the treatment of MS and diabetes [22, 23]. Interestingly, the G6P excess in the liver ER has been associated to increased 11βHSD1 activity in GSDIa [2]. The increased 11βHSD1 activity might play a role in the increased prevalence of insulin-resistance (IR) and MS reported in GSDIa patients [24].

Biochemically, glucocorticoid synthesis involves the shuttling of precursors between mitochondria and the ER, with cholesterol entering the mitochondria as first step [25]. Most steroidogenic cholesterol is derived from circulating lipoproteins, but it may be also produced de novo within the ER [26]. Interestingly, increased G6P levels in ER [27] and mitochondrial dysfunction [28] have been suggested to be the cause and the effect of hypercholesterolemia in GSDIa, respectively. Notably, G6Pase activity has been shown in zona reticularis and zona fasciculata that are actively involved in cortisol synthesis [29]. The increase of cortisol synthesis might in principle represent a mechanism to divert cholesterol excess within the mitochondria in GSDIa. Correlation data support this hypothesis. Despite not statistically significant, these data suggest that the combination of cholesterol and TG would best explain the cortisol levels in GSDI patients. The lack of significance at multivariate analysis might be due to small sample size and high correlation between the two independent variables.

GSDIb is typically associated with neutropenia, neutrophil dysfunction and predisposition to inflammatory bowel disease (IBD) [1]. Increased prevalence of autoimmune disorders has been reported [10, 30]. In GSDIb the lack of G6P in ER has been associated to decreased 11βHSD1 activity [2]. 11βHSD1 is widely expressed in immune cells [31]. 11βHSD1 expression has been associated with a switch in energy metabolism suggesting that 11βHSD1 deficiency might worsen tissue damage in the case of chronic inflammation [32, 33]. Indeed, 11βHSD1-deficient mice showed delayed resolution of the inflammation [34]. Glucocorticoids are also essential regulators of T-cells development [35]. The engagement of glucocorticoid receptor has been recently shown as crucial determinant conferring protection from autoimmunity during pregnancy in mice [36]. Regulatory T cells (Tregs) are particularly responsive to glucocorticoid signals [37] and impairment of Tregs has been described in a number of autoimmune diseases [38]. Interestingly, disrupted Tregs function has been reported in GSDIb patients [39]. We hypothesize that reduced 11βHSD1 activity in GSDIb patients’ immune cells could impair energy metabolism and cell function and play a role in delayed resolution of inflammation and development of autoimmune disorders.

## Conclusions

Opposite cortisol levels were found in GSDIa (increased) and GSDIb (decreased) patients. The findings of the current study suggest that imbalanced cortisol concentrations might be due to local deregulation rather than HPA axis activation in GSDI. 11βHSD1 activity modulation by G6P availability could explain the opposite cortisol profile in GSDIa and GSDIb patients. We speculate that glucocorticoid deregulation might play a role in the development of the emerging complications in GSDIa (namely IR and MS) and GSDIb (delayed inflammation, autoimmune disorders) patients (Fig. 1). The results of the current study suggest that adrenal evaluation should be considered to define the pathophysiology of complications in GSDI and possibly provide additional disease biomarker. It is noteworthy that the dysregulation of cortisol secretion is opposite in GSDIa and GSDIb. Future studies dissecting the connection between G6Pase system and 11βHSD1 are warranted in order to identify new potential therapeutic targets in GSDI patients.

## Supplementary information

**Additional file 1 **Biochemical and baseline adrenal cortex hormones in GSDIa patients (●), GSDIa-related controls (■), GSDIb patients (▲) and GSDIb-related controls (◆) *p < 0.05, **p < 0.01, ***p < 0.001.

**Additional file 2 **Cortisol (●) and glucose (■) concentrations at the beginning and at the end of the ACTH stimulation test in GSDIa (A,B,C) and GSDIb (D) patients. *T30: 30 min after ACTH analogue administration, T60: 60 min after ACTH analogue administration, T90: 90 min after ACTH analogue administration.*

## Data Availability

The datasets used and/or analysed during the current study are available from the corresponding author on reasonable request.

## Abbreviations

ER: Endoplasmic reticulum
G6P: Glucose 6-phosphate
G6Pase: Glucose-6-phosphatase
11βHSD1: 11β-hydroxysteroid dehydrogenase type 1
HPA axis: Hypothalamo-Pituitary-Adrenal Axis
