# Supplementary material for: Imbalanced cortisol concentrations in glycogen storage disease type I: evidence for a possible link between endocrine regulation and metabolic derangement
Source: Orphanet J Rare Dis. 2020 Apr 19;15:99. doi: 10.1186/s13023-020-01377-w (PMC7169016; doi:10.1186/s13023-020-01377-w)
Supplement: Supplementary file 1 — Additional file 1 Biochemical and baseline adrenal cortex hormones in GSDIa patients (●), GSDIa-related controls (■), GSDIb patients (▲) and GSDIb-related controls (◆) *p < 0.05, **p < 0.01, ***p < 0.001. [file 13023_2020_1377_MOESM1_ESM.docx]

**Additional file 1.** Biochemical and baseline adrenal cortex hormones in GSDIa patients ( ), GSDIa-related controls ( ), GSDIb patients ( ) and GSDIb-related controls ( ) *p< 0.05, **p<0.01, ***p<0.001
