# Supplementary material for: Imbalanced cortisol concentrations in glycogen storage disease type I: evidence for a possible link between endocrine regulation and metabolic derangement
Source: Orphanet J Rare Dis. 2020 Apr 19;15:99. doi: 10.1186/s13023-020-01377-w (PMC7169016; doi:10.1186/s13023-020-01377-w)
Supplement: Supplementary file 2 — Additional file 2 Cortisol (●) and glucose (■) concentrations at the beginning and at the end of the ACTH stimulation test in GSDIa (A,B,C) and GSDIb (D) patients. T30: 30 min after ACTH analogue administration, T60: 60 min after ACTH analogue administration, T90: 90 min after ACTH analogue administration. [file 13023_2020_1377_MOESM2_ESM.docx]

**Additional file 2.** Cortisol ( ) and glucose ( ) concentrations at the beginning and at the end of the ACTH stimulation test in GSDIa (A,B,C) and GSDIb (D) patients. *T30: 30 minutes after ACTH analogue administration, T60: 60 minutes after ACTH analogue administration, T90: 90 minutes after ACTH analogue administration*
